# 

**Published:** 1852-06

**Authors:** 


### CONTENTS.

ORIGINAL COMMUNICATIONS :— PAGE.

ART. 1.—On the Pathology of Spontaneous Hæmorrhage, by A. B. Palmer, M.D. - - - - - - - - - - - - 49
ART. 2.—On the Uncertainty of Diagnosis by Physical Signs in the first stages of Diseases of the Chest, by J. W. Freer, M.D., - - - - - 56
ART. 3.—Vegetable Diet in the Chronic Diarrhœa of California, by S. R. Haven, M.D., - - - - - - - - - - - - - - 61

SELECTIONS :—

The Liver and its Secretions, - - - - - - - - - 63
Abstract of Proceedings of American Medical Association, - - - 65
Opium, - - - - - - - - - - - - 71
On Blistering in Gleet, - - - - - - - - - - - 73
Sinapisms to the Mammæ for Suppression of the Menses, - - - 75
Prophylaxis of Epidemic of Puerperal Fever, - - - - - - - 76
Calomel and Soda as a Cathartic, - - - - - - - - - 77
Tobacco Smoke in Strangulated Hernia, - - - - - - - 78
Quinine in Typhus and Typhoid Fevers, - - - - - - - 79
Saline Injections for the Cure of Drunkenness, - - - - - - 80
Turpentine in Dysentery, - - - - - - - - - - 80

EDITORIAL :—

Beck's Lectures on Materia Medica and Therapeutics, - - - - - 81
The Medical Student's Vade Mecum, - - - - - - - - 84
The East Tennessee Record of Medicine and Surgery, - - - - 85
Reese's Analysis of Physiology, - - - - - - - - - 86
Review of Materia Medica for the use of Students, - - - - - 86
Cook County Medical Society, - - - - - - - - - 87
Meeting of the Illinois State Medical Society, - - - - - - 90
Semi-Annual Meeting of the Æsculapian Society at Palestine, - - 92
The "Med. Department of the University of Iowa," and the Am. Med. Ass., 94
Achromatic Microscopes, - - - - - - - - - - 95
Obituary, - - - - - - - - - - - - - 96
Tartaric Acid used for aiding the solution of Di-Sulphate of Quinia. - 96

